# Prognostic factors of myringoplasty: study of a 140 cases series and review of the literature

**DOI:** 10.11604/pamj.2019.33.323.18060

**Published:** 2019-08-26

**Authors:** Youssef Darouassi, Abdelfettah Aljalil, Amine Ennouali, Mohamed Amine Hanine, Youness Chebraoui, Brahim Bouaity, Mohamed Mliha Touati, Haddou Ammar

**Affiliations:** 1 ENT Department Military Hospital Avicenna, Marrakech, Morocco

**Keywords:** Myringoplasty, surgery, tympanic membrane perforation

## Abstract

Myringoplasty is one of the most frequent interventions in otology. It aims to restore the eardrum in order to protect against extrinsic contamination by water and to improve hearing. Our study aimed to analyze the factors that may affect anatomical and functional results of myringoplasty or type I tympanoplasty. A retrospective study was performed of a series of 140 cases of myringoplasty over a 6-years period from 2010 to 2015. The approach was post-auricular in 69% of cases and all the patients underwent an underlay technique. Temporal fascia was used in 90.71% of the cases. After an average follow-up of 13 months, the anatomical and functional results were acceptable, with a tympanic closure rate of 88% and an average audiometric gain of 14.22 dB. Several factors affected our results, including the location of the perforation, the active or inactive status of the chronic otitis media, the condition of the opposite ear and the graft material. In light of our results and those of the literature, we believe that the middle ear should be dry at least two months prior to surgery, use of cartilaginous graft material and underlay technique should be preferred and special precautions should be taken in case of anterior or contralateral perforation.

## Introduction

Myringoplasty, or type 1 tympanoplasty, is one of the most frequent interventions in otology. It aims to restore the eardrum in order to protect against extrinsic contamination by water and to improve hearing. Several prognostic factors that may affect the outcome of myringoplasty has been reported in the literature. The incidence of surgical success of tympanoplasty ranges from 60% to 99% in adults [1]. The main objective of our study is to analyze, in light of our results and a review of the literature, the factors that may affect the anatomical and functional results of this surgery.

## Methods

We performed a retrospective study of a series of 140 cases of myringoplasty performed in our department during the period from 2010 to 2015. We included all patients operated for simple tympanic perforation. We excluded cases with cholesteatoma and with incomplete data. All patients were operated using microscope. We analyzed clinical records, surgery reports, audiograms and results at follow-up taking into account the graft integration and the average audiometric gain. The collection of data was carried on without compromising the anonymity of the patients and the confidentiality of their information.

## Results

**Figure 1:**
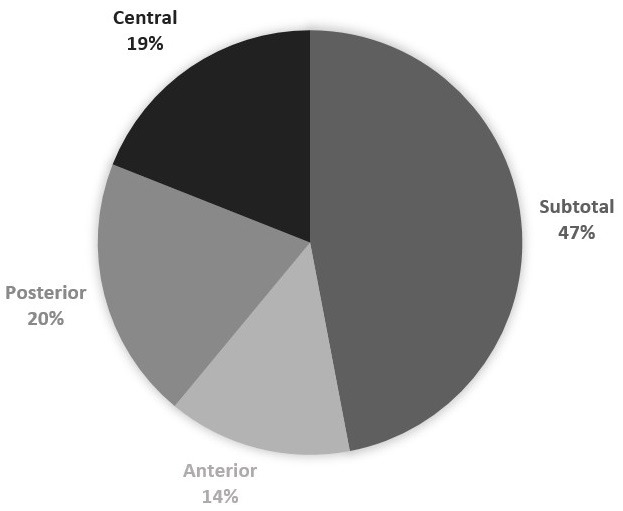
Distribution in our series according to the location of the perforation

The age of the patients ranged from 8 to 75 years with an average of 34 years. The sex ratio was 1.18. Twenty-one patients had bilateral chronic otitis media. The perforation was subtotal in 65 cases (47%), anterior in 20 cases (14%), posterior in 28 cases (20%) and central in 27 cases (19%) (Figure 1). The middle ear mucosa was dry at the time of surgery in all cases. Pure-tone audiometry showed conductive hearing loss in 121 patients (86%) and mixed hearing loss in 19 cases (14%). The average preoperative air-bone gap was 30.38 dB. The intervention was primary in 136 cases. Four patients underwent a revision surgery within one year after primary surgery and eight patients underwent bilateral myringoplasty. Surgical approach was post-auricular in 97 cases (69%) and endaural in 43 cases (31%). The graft was temporalis fascia in 90.71% of cases, temporalis fascia with cartilage in 7.14% of cases and cartilage only in three cases. We used the underlay technique for placing the graft in all cases. The duration of follow-up ranged from 1 month to 46 months with an average of 13 months, but 27 patients were lost to follow-up. Among the 113 remaining cases, 99 patients (88%) had a successful graft integration. We observed an improvement in hearing in 95 cases (84.07%). Distribution of the patients according to postoperative audiometric gain is showed in Table 1. The average audiometric gain was 14.22 dB. Air-bone gap remained unchanged in nine cases (7.96%) and declined in eight cases (7.07%). The graft integration rate was 84.62% for subtotal perforations, 95.45% for central perforations, 95.65% for posterior perforations but only 75% for anterior perforations (statically significant). The closure rate was 89.05% in case of a normal contralateral ear and only 62% if it was diseased. With regard to the graft material, the closure rate was 87.25% for temporal fascia, 87.5% for fascia with cartilage and 100% for cartilage only. When the temporal fascia was used, the average postoperative air-bone gap was <20 dB in 51% of cases, between 20 and 30 dB in 31.85% of cases and greater than 30 dB in 10, 61% of cases. While in the three cases where the cartilage was used, the audiometric gain was 10 dB.

**Table 1:** Distribution in our series according to postoperative audiometric gain

Postoperative audiometric gain	Number of case	Percentage
01-09 dB	15	15.79%
10-15 dB	50	52.63%
15-25 dB	19	20%
25-30 dB	11	11.58%

## Discussion

Myringoplasty is one of the most frequent procedures in otology because of the high incidence of tympanic membrane perforations. A preoperative pure-tone audiometry is required to assess the hearing loss, to choose the side to start with and to compare with post-operative functional results [2]. Chronic otitis media usually causes conductive hearing loss ranging from 30 to 50 dB. The average preoperative air-bone gap in our study was 30.38 dB. Surgical approach may be posterior, endaural or permeatal. The choice is conditioned by the size of the external auditory meatus and the location of the perforation [3]. In the other hand, endoscopic myringoplasty has several advantages [4]. Several graft materials may be used: temporalis fascia, perichondrium, cartilage, fat, areolar tissue, alloderm, vein, and basic fibroblast growth factor [5,6]. The graft can be placed either laterally (overlay) or medially (underlay) to the fibrous layer. Each of the two techniques have its indications, advantages and disadvantages. However, there is much support for the underlay technique: ease, shorter surgical procedure, easier assessment of the mobility of the ossicular chain, faster healing, better hearing results and fewer complications [6].

There are divergences in defining the success of the intervention. Several authors retain the graft integration. Others add the absence of atelectasis, graft lateralization and anterior-angle filling; and on the functional level: normal hearing, hearing gain of at least 25 dB, air-bone gap closure of 15 dB or an average residual air-bone gap less than 10 dB [7-11]. The graft integration rate of myringoplasty varies from 60% to 99% [1]. In our series, 88% of followed-up patients had a tympanic perforation closure. Several factors can impact the result such as the disease, the environment (smoking), the surgeon's experience, the surgery indication, the choice of graft material and its positioning [12]. According to many authors [4,13,14], anterior location have a poor prognosis, probably due to poor vascularization and more difficult access [6]. This is consistent with our results. However, other studies [1,15] found no impact of the location of the perforation. The size of the perforation affected the success rate in our series and in many studies [4,16,17] but had no impact in other ones [13,18]. Usually, myringoplasty is performed in case of inactive otitis media with a normal mucosa, but this is almost impossible to achieve in many cases despite medication. Dry middle ear status of less than two months at the time of surgery is an independent prognosis factors [19]. However, active otitis media status with inflamed mucosa did not affect the results in some studies [17,20]. The condition of the opposite ear had affected our results. A closure rate of 89.5% was observed when the contralateral ear was normal compared to 62% when it was diseased, in agreement with certain authors [16,21]. Others [22,23] did not find any impact.

The most frequently used graft material is the temporalis fascia. Some authors [3,24] proposed the temporal fascia as a graft material in all patients. However, the cartilage has been believed to be more appropriate since it is more rigid and more resistant to infection, resorption and retraction [25] with a higher closure rate in many studies [26,27], while others [28] noted the opposite. In our study, the temporal fascia was the most commonly used graft material with a closure rate of 87.25%; cartilage was used alone in only three cases with a 100% anatomical success. According to Kallel *et al.* [14], the postoperative air-bone gap variation was statistically related to the size of the perforation. The graft material is also considered as a factor affecting the audiometric results [28-30], while some authors [8] found comparable outcomes. In our study, in the cases where the temporal fascia was used, the average postoperative air-bone gap was less than 20 dB in 51%, between 20 and 30 dB in 31.85% of cases and greater than 30 dB in 10,61% of cases whereas our 3 patients who benefited from a cartilaginous graft had a 10 dB audiometric gain.

## Conclusion

Myringoplasty is the one of the most commonly performed procedures in otology. The underlay technique and temporal fascia remain the most commonly used. Many factors affected our anatomical and functional results notably the location of the perforation, the dry middle ear condition and the condition of the opposite ear. Given our results and those of the literature, we recommend to use the underlay technique and to take special attention in case of anterior or contralateral perforation. We believe that the middle ear mucosa should be dry at the time of surgery for at least two months.

### What is known about this topic

- Myringoplasty is one of the most performed surgeries in otology;
- Surgical success of myringoplasty ranges from 60% to 99% in adults;
- Several factors may influence the prognosis of myringoplasty.

### What this study adds

- Several divergences persist between the authors on the definition of the success of the surgery and on the prognostic factors;
- We recommend to use the underlay technique and to take special attention in case of anterior or contralateral perforation;
- We believe that the middle ear mucosa should be dry at the time of surgery for at least two months.

## Competing interests

The authors declare no competing interests.
